# Properties of Longitudinal Electromagnetic Oscillations in Metals and Their Excitation at Planar and Spherical Surfaces

**DOI:** 10.1186/s11671-017-2230-6

**Published:** 2017-08-01

**Authors:** Vitaly V. Datsyuk, Oleg R. Pavlyniuk

**Affiliations:** Department of Physics, Taras Shevchenko National University of Kyiv, 64, Volodymyrska Str., 01601 Kyiv, Ukraine

**Keywords:** Nonlocal electrodynamics, Lindhard dielectric function, Surface plasmon resonance

## Abstract

The common definition of the spatially dispersive permittivity is revised. The response of the degenerate electron gas on an electric field satisfying the vector Helmholtz equation is found with a solution to the Boltzmann equation. The calculated longitudinal dielectric function coincides with that obtained by Klimontovich and Silin in 1952 and Lindhard in 1954. However, it depends on the square of the wavenumber, a parameter of the vector Helmholtz equation, but not the wave vector of a plane electromagnetic wave. This new concept simplifies simulation of the nonlocal effects, for example, with a generalized Lorents–Mie theory, since no Fourier transforms should be made. The Fresnel coefficients are generalized allowing for excitation of the longitudinal electromagnetic waves. To verify the theory, the extinction spectra for silver and gold nanometer-sized spheres are calculated. For these particles, the generalized Lorents–Mie theory gives the blue shift and broadening of the plasmon resonance which are in excellent agreement with experimental data. In addition, the nonlocal theory explains vanishing of the plasmon resonance observed for gold spheres with diameters less than or equal to 2 nm. The calculations using the Klimontovich-Silin-Lindhard and hydrodynamic dielectric functions for silver are found to give close results at photon energies from 3 to 4 eV. We show that the absolute values of the wavenumbers of the longitudinal waves in solids are much higher than those of the transverse waves.

## Background

Irradiation of a plane metal surface by femtosecond laser pulses often results in formation of laser-induced periodic surface structures (LIPSSs) [1]. Besides the LIPSS, hyperfine ripples called high-spatial-frequency LIPSS (HSFL) were observed [1, 2]. The spatial periods of the HSFL are significantly smaller than the irradiation wavelength *λ*_0_. For example, for aluminum this period was estimated to range from 20 to 200 nm at *λ*_0_=0.8 *μ*m [2, 3]. While orientation of the ripples in ordinary LIPSS was perpendicular to the laser light polarization, the orientation of HSFL was often perpendicular and sometimes parallel to the polarization. Similar HSFL were formed on the surfaces of transparent dielectrics, semiconductors, and metals. The origin of the HSFL was explained by different mechanisms such as second-harmonic generation, the involvement of specific types of plasmon modes, self-organization, and local field enhancements during inhomogeneous breakdown in dielectric materials [2, 3].

The goal of this study is to search a wave process which could produce a pattern with a short period *Λ*≪*λ*_0_. We examine properties of longitudinal (L) electromagnetic waves in metals also known as plasma waves. Our study consists of the following novel steps. First, we started our research with definition of the spatial dispersion of the permittivity. As shown below, the common definition is useless if a medium under study is not uniform and infinite. Therefore, we propose a new concept of the spatially dispersive dielectric function *ε*. This function establishes the direct proportionality between two vector fields, **E**(**r**,*ω*) and **D**(**r**,*ω*), but not the amplitudes **E**(**k**,*ω*) and **D**(**k**,*ω*) of plane waves. Consequently, the quantity *ε* depends on the square of the wave number, *k* ^2^, the parameter of the vector Helmholtz equation for the electric field **E**(**r**,*ω*), but not the wave vector **k** of the plane waves. Then, to derive such a novel function, we determined the response of the conduction electrons on an electromagnetic mode by solving the Boltzmann transport equation written in the relaxation-time approximation. The so-called transverse and longitudinal Lindhard dielectric functions were obtained. Further, we found that the longitudinal Lindhard and much simpler hydrodynamic function are close in a wide range of parameters. Light extinction by silver and gold nanospheres was considered in order to illustrate the theory. We show for the first time that the nonlocal Mie theory explains the blue shift, broadening, and eventual vanishing of the plasmon resonances observed with a decrease of the size of the noble-metal nanospheres. Finally, the newly developed theoretical model was applied to examine the possibility of involvement of the longitudinal modes in formation of the laser-induced surface structures. For this purpose, we modified the Fresnel theory taking into account transmitted longitudinal waves.

## Methods

To determine the electromagnetic fields in piecewise homogeneous media, the classical electromagnetic theory was applied. The electric field **E** in each uniform domain of the heterogeneous medium was assumed to be a solution of the vector Helmholtz equation (VHE):

1 $$  \Delta\,\mathbf{E} + k^{2}\, \mathbf{E}=0,  $$

where *Δ* is the Laplace operator.

As usual, the tangential components of the electric **E** and magnetic **H** fields are continuous across the boundaries of the media. In addition, we took into account that electrons are confined in metal; therefore, the following additional boundary condition (ABC) for the normal component of the current density **j** at the metal surface *S* was used: (**j** **n**)|_**r**∈*S*_=0.

To determine the conduction current in metal, we solved the Boltzmann transport equation (BTE) written in the relaxation time approximation:

2 $$  \frac{\partial f}{\partial t}+\mathbf{v}\,\frac{\partial f}{\partial \mathbf{r}}+ \frac{e}{m}\,\left(\mathbf{E}+\mathbf{v}\times\mathbf{B} \right)\,\frac{\partial f}{\partial \mathbf{v}} = \frac{f_{0}-f}{\tau},  $$

where *f* is the single-particle distribution function in the phase space (**r**, **v**), **v** is the microscopic electron velocity, *e* and *m* are the electron charge and mass respectively, **B** is the magnetic induction, *f*_0_ is an equilibrium distribution function, and *τ* is the relaxation time.

Below, we derive formulas for the spatially dispersive dielectric functions. Then, we use them to study light reflection from a plane metal surface and scattering of light on a noble-metal nanosphere.

## Results and Discussion

### Spatial Dispersion of *ε* in a Heterogeneous Medium

In the literature, a spatially dispersive dielectric function *ε* is defined via the following relation [4–6]:

3 $$  \mathbf{D}(\omega,\, \mathbf{r}) = \epsilon_{0} \iiint\limits_{-\infty}^{\infty} \! \mathbf{d} \mathbf{r}^{\prime}\, \epsilon\left(\omega, \, \mathbf{r}-\mathbf{r}^{\prime}\right)\,\mathbf{E}\left(\omega,\, \mathbf{r}^{\prime}\right),  $$

where *ε*_0_ is the electric constant, **D**(*ω*, **r**) is the amplitude of the displacement vector oscillation with angular frequency *ω* in point **r**, and **E**(*ω*, **r** ^′^) the amplitude of the electric-field oscillation in point **r** ^′^. Fourier transforms of Eq. (3) give the equation

4 $$ \mathbf{D}(\omega,\, \mathbf{k}) = \epsilon_{0} \, \epsilon(\omega,\, \mathbf{k})\,\mathbf{E}(\omega,\, \mathbf{k})  $$

where a spatially dispersive *ε*(*ω*, **k**) depends on the wave vector **k** of a plane electromagnetic wave. In our opinion, Eq. (3) is not ambiguous only in an infinite homogeneous volume but we deal with piecewise heterogeneous system where boundaries should be taken into account and **k** are not the same in different media.

Our approach does not use expansion of the electromagnetic waves over plane waves. The spatially dispersive permittivity determines the relation between **D**(*ω*, **r**) and a particular solution to the vector Helmholtz Eq. (1):

5 $$  \mathbf{D}(\omega,\, \mathbf{r}) = \epsilon_{0} \, \epsilon(\omega, \, k)\,\mathbf{E}(\omega,\, \mathbf{r}).  $$

Here **E**(*ω*, **r**) denotes distribution of the electric field but not merely the vector **E** in point **r**.

### Longitudinal and Transverse Dielectric Functions

The permittivity of metals is commonly expressed through the conductivity *σ* [4]:

6 $$  \epsilon=\epsilon_{\mathrm{g}}+\frac{i\,\sigma}{\omega\,\epsilon_{0}},  $$

where *ε*_g_ is a part of the dielectric function allowing for polarization of the solid; *ε*_g_=1 for a simple metal. In order to determine *σ*, we calculated the current density

7 $$  \mathbf{j}= e \iiint\limits_{-\infty}^{\infty} \! \mathbf{v}\, f\, \mathrm{d}\/ \mathbf{v}=\sigma\,\mathbf{E},  $$

where $\mathrm {d} \mathbf {v}= \frac {v}{m}\,\mathrm {d} \epsilon \,\mathrm {d}\,\Omega,$ d *Ω*= sin*θ*d*θ* d*ϕ*, *v*, *θ*, *ϕ* are the spherical coordinates of the velocity. Unlike previous researches, we did not introduce the wave vector **k** but found a BTE solution in a form of an infinite series containing operators **v** **∇** acting on **v** **E** :

8 $$ f=f_{0} + \frac{e}{-i \omega+\Gamma}\,\frac{\partial f_{0}}{\partial \epsilon} \left[ 1+\frac{\mathbf{v}\,{\mathbf{\nabla}}}{-i \omega+\Gamma} \right]^{-\,1}\! \mathbf{v}\,\mathbf{E},  $$

where *Γ*=1/*τ*. Then, *f*_0_ was approximated by a zero-temperature Fermi-Dirac distribution and, after integration over *ε* in Eq. (7), we got

9 $$ \mathbf{j}=\frac{\omega_{\mathrm{p}}^{2}\,\varepsilon_{0}}{-i \omega+\Gamma}\,\frac{3}{4 \pi} \iint \mathbf{u}\,\left(1+ l\,\mathbf{u} \mathbf{\nabla} \right)^{-\,1} (\mathbf{u}\,\mathbf{E})\, \mathrm{d}\,\Omega,  $$

where $\omega _{\mathrm {p}}^{2}= \frac {e^{2}\,n_{e}}{m\,\varepsilon _{0}},$ *ω*_p_ is the plasma frequency, $\mathbf {u}=\frac {\mathbf {v}}{v}$ is the unit vector in the direction of **v**,$l= \frac {v_{\mathrm {F}}}{-i \omega +\Gamma },$ *v*_F_ is the Fermi velocity. Further, we calculated the integrals

10 $$\begin{array}{*{20}l} &\textstyle \iint \mathbf{u}\, (\mathbf{u}\,\mathbf{E})\,\mathbf{d}\,\Omega = \frac{4\/\pi}{3}\,\mathbf{E}  \end{array} $$

11 $$\begin{array}{*{20}l} &\textstyle \iint \mathbf{u}\,(\mathbf{u}\,\mathbf{\nabla})^{2 n-1} (\mathbf{u}\,\mathbf{E})\,\mathbf{d}\,\Omega = 0  \end{array} $$

12 $$\begin{array}{*{20}l} &\textstyle \iint \mathbf{u}\,(\mathbf{u}\,\mathbf{\nabla})^{2 n} (\mathbf{u}\,\mathbf{E})\,\mathbf{d}\,\Omega = \frac{4 \pi}{2 n+3} \\ &\times \, \Delta^{n-1} \left[ \mathbf{\nabla}\, (\mathbf{\nabla}\cdot\mathbf{E})-\frac{1}{2 n+1}\, \mathbf{\nabla}\times \mathbf{\nabla}\times\mathbf{E}\right]  \end{array} $$

where *n* is a natural number. The following dependence of **j** on an *arbitrary* electric field **E** was finally obtained

13 $$\begin{array}{*{20}l}  \mathbf{j}&=\frac{\omega_{\mathrm{p}}^{2}\,\varepsilon_{0}}{-i \omega+\Gamma} \left\{\mathbf{E} + 3\, \sum\limits_{n=1}^{\infty} l^{\,2 n} \right. \\ &\quad\left.\times \frac{\Delta^{n-1}}{2 n+3} \left[ \mathbf{\nabla}\, (\mathbf{\nabla}\cdot\mathbf{E})-\frac{\mathbf{\nabla}\times \mathbf{\nabla}\times\mathbf{E}}{2 n+1}\right]\right\}. \end{array} $$

There are two types of solutions to Eq. (1), divergence-free which satisfy equation **∇**·**E**=0 and rotationless which satisfy equation

14 $$  \mathbf{\nabla} \times \mathbf{E}=0.  $$

For a plane wave, with *E*∝ exp[*i* (**k** **r**−*ω* *t*)], Eq. (14) transforms into the relation **k**×**E**=0 which shows that the wave is longitudinal (L). To simulate processes in spherical bodies, it is convenient to use the vector spherical harmonics **L**, **M**, and **N** as a complete set of orthogonal functions. In this case, Eq. (14) specifies harmonics **L**. The wavenumber of the L waves and **L** modes is determined by the following dispersion law

15 $$  \epsilon^{\mathrm{L}}\left(\omega, \, k^{\mathrm{L}}\right)=0.  $$

From Eqs. (6) and (13) we find that solutions to Eq. (1) satisfying the constraint of Eq. (14) give the following longitudinal permittivity

16 $$  \epsilon^{\mathrm{L}}=\epsilon_{\mathrm{g}}- \frac{\omega_{\mathrm{p}}^{2}}{\omega\,(\omega+i \Gamma)}\,\frac{3}{2} \, \Phi \left(a^{2},\, 1,\,\frac{3}{2} \right)  $$

where *Φ* is the Lerch’s Phi function,

17 $$ \frac{3}{2} \, \Phi \left(a^{2},\, 1,\,\frac{3}{2} \right) =\sum\limits_{n=0}^{\infty} \frac{3}{2 n+3}\, a^{2\,n},  $$

$a=\frac {k v_{\mathrm {F}}}{\omega +i \Gamma }$.

The obtained permittivity differs from that defined by Kliewer and Fuchs [7] only in notation:

18 $$ \epsilon^{\mathrm{L}}=\epsilon_{\mathrm{g}}+ \frac{\omega_{\mathrm{p}}^{2}}{\omega\,(\omega+i \Gamma)}\,\frac{3}{a^{2}}\left[1-\frac{1}{i a} \tan^{-1}(i a) \right]   $$

The identity

19 $$ \frac{1}{i a}\tan^{-1}(i a)=\frac{1}{2}\ln\frac{1+a}{1-a}  $$

allows one to rewrite Eq. (18) as follows

20 $$ \epsilon^{\mathrm{L}}=\epsilon_{\mathrm{g}}- \frac{\omega_{\mathrm{p}}^{2}}{\omega\,(\omega+i \Gamma)}\,\frac{3}{a^{2}}\left[1-\frac{1}{2 a} \, \frac{\ln(1+a)}{\ln(1-a)} \right].   $$

In the case of *Γ*=0, this formula takes the form of an equation derived by Klimontovich and Silin [8] who studied Landau dumping in degenerate plasma (see [9], [10, Eq. (40.17)], and [11]). The permittivity of the equivalent Eqs. (16), (18), and (20) is commonly called the Lindhard dielectric function (with reference to [12]) though this function was first obtained by Klimontovich and Silin [8].

The transverse Lindhard permittivity [7] can be found with Eq. (13) when **∇**·**E**=0. In the actual case of *v*_F_ *k*≪*ω*, it reduces to the Drude dielectric function

21 $$  \epsilon^{\mathrm{T}}=\epsilon_{\mathrm{g}}- \frac{\omega_{\mathrm{p}}^{2}}{\omega^{2}+i\,\Gamma\/\omega}.  $$

This function agrees with experimental data on many metals [13]. If |*a*|<1, the longitudinal permittivity (16) simplifies to the hydrodynamic dielectric function:

22 $$  \epsilon^{\mathrm{L}}\left(\omega,\,k^{\mathrm{L}}\right)=\epsilon_{\mathrm{g}}- \frac{\omega_{\mathrm{p}}^{2}}{\omega^{2}+i\,\Gamma \omega-\frac{3}{5}\,\left(v_{\mathrm{F}}\, k^{\mathrm{L}}\right)^{2}}.  $$

### Reflection of a Plane Electromagnetic Wave from a Flat Metal Surface

#### Boundary Conditions

In this section, we determine the direction of the wave vector *k* ^L^ and amplitude of the L wave excited in metal during reflection of a plane electromagnetic wave from a flat metal surface.

Consider plane wave incident on the dielectric-metal interface *z*=0 with the wave vector lying in the *xz* plane. The electric field in the dielectric medium 1 consists of the incident **E**_i_ and reflected **E**_r_ waves, the field in metal 2 has the transmitted transverse **E**_t_ and, in some cases, longitudinal **E** ^L^ components. According to the Maxwell boundary conditions, the transverse components of the electric and magnetic field vectors are continuous in plane *z*=0. In addition, the electrons are not ejected from metal; therefore, the normal component of the electric current density is zero at *z*=0,

23 $$ \hat{\mathbf{z}}\,\mathbf{j}|_{z=0}=0.   $$

were $\hat {\mathbf {z}}$ is the unit vector in the direction of *z* axis.

All terms in the Maxwell boundary conditions must have the same dependence on *x* and *y*. This requirement has several consequences. First, it can be established that L waves can be excited only in the case of p-polarization when the electric vector of the incident wave **E** ^(*i*)^ is parallel to the plane of incidence. In other words, plasmon polaritons can be generated by a transverse magnetic (TM) wave. The effect is much the same as in a metal sphere [14]. Secondly, formulas akin to the Snell’s law can be derived from the conditions

24 $$  k_{1x}=k_{2x}=k_{2x}^{\mathrm{L}}=k_{1}\,\sin\theta_{1}  $$

where indexes 1*x* and 2*x* denote the *x*-projections of the vectors in media 1 and 2, respectively, *θ*_1_ is the angle of incidence.

#### Reflection and Transmission Coefficients

Let us determine the field formed by a plane p-polarized electromagnetic wave incident on a plane metal surface. It is convenient to express the components of the electric and magnetic fields through the *x* component of **E** ^(*i*)^, namely *E* *x*(r)=− *r* *E* *x*(i) for the reflected wave, *E* *x*(t)=*t* *E* *x*(i) for the transmitted transverse wave, and

25 $$  E^{\mathrm{(a)}}_{x}=\delta\,E^{\mathrm{(t)}}_{x}=t_{\mathrm{L}}\,E^{\mathrm{(i)}}_{x}  $$

for the transmitted longitudinal wave, here *r* is a reflection coefficient, *t* and *t*_L_ are transmission coefficients.

From the Maxwell boundary conditions and ABC of Eq. (23) written in the following form

26 $$ \hat{\mathbf{z}}\,(\mathbf{D}-\epsilon_{0}\epsilon_{\mathrm{g}}\,\mathbf{E})|_{z=0}=0,  $$

we got

27 $$\begin{array}{*{20}l} r&=- \frac{(1+\delta)\,\epsilon_{1}\,k_{2z}-\epsilon_{2}\,k_{1z}}{(1+\delta)\,\epsilon_{1}\,k_{2z}+\epsilon_{2}\,k_{1z}}=1-(1+\delta)\,t  \end{array} $$

28 $$\begin{array}{*{20}l} t&=\frac{2\,\epsilon_{1}\,k_{2z}}{\epsilon_{2}\,k_{1z}+(1+\delta)\,\epsilon_{1}\,k_{2z}}, \end{array} $$

29 $$\begin{array}{*{20}l} \delta&=\frac{\epsilon_{\mathrm{g}}-\epsilon}{\epsilon_{\mathrm{g}}}\,\frac{k_{2x}^{2}}{k_{2z}\,k_{2z}^{\mathrm{L}}} \end{array} $$

At *δ*=0, the coefficient *r* becomes the Fresnel coefficient of reflection of the p-polarized wave (see, for instance, Eq. (2.49) of [4]). Under the same condition, *t* is not the Fresnel transmission coefficient since our definitions of *t* and *r* differ from the Fresnel’s ones.

### Extinction of Light by Metal Nanosphere

In a preceding paper, one of the authors generalized the Lorentz-Mie theory allowing for the ABC of Eq. (23). An analog of the Fresnel coefficient *r*, the Mie coefficient *b*_*l*_ for the reflected TM mode of the *l*th order was found to be

30 $$  b_{l}=- \frac{(1+\delta_{l})\,\epsilon_{1}\, \frac{k_{2}\,\psi_{l}^{\prime}(k_{2} R)}{\psi_{l}(k_{2} R)} - \epsilon_{2}\, \frac{k_{1}\,\psi_{l}^{\prime}(k_{1} R)}{\psi_{l}(k_{1} R)}}{(1+\delta_{l})\,\epsilon_{1}\, \frac{k_{2}\,\psi_{l}^{\prime}(k_{2} R)}{\psi_{l}(k_{2} R)} - \epsilon_{2}\, \frac{k_{1}\,\zeta_{l}^{\prime}(k_{1} R)}{\zeta_{l}(k_{1} R)}},  $$

where

31 $$ \delta_{l}=\frac{\epsilon^{\mathrm{T}}- \epsilon_{\mathrm{g}}}{\epsilon_{\mathrm{g}}}\, \frac{l\,(l+1)\,j_{l}(k_{2} R)\,j_{l}(k_{2}^{\mathrm{L}} R)}{\psi_{l}^{\prime}(k_{2} R)\,k_{2}^{\mathrm{L}} R \,j_{l}^{\prime}\left(k_{2}^{\mathrm{L}} R\right)},  $$

*ψ*_*l*_ and *ζ*_*l*_ are the Riccati-Bessel and Riccati-Hankel functions of the order *l*, respectively; *j*_*l*_ is the spherical Bessel function, the prime denotes the derivative of a function with respect to its argument.

**Fig. 1:**
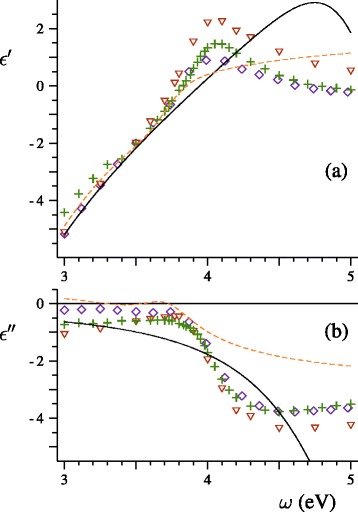
Real (**a**) and imaginary (**b**) parts of the dielectric function of silver according to Johnson and Christy (◇) [20], Lynch and Hunter (+) [16], Weber (△) [21], Hao and Nordlander (*dashed line*) [22], and Drachev et al. (*solid line*) [23]

**Fig. 2:**
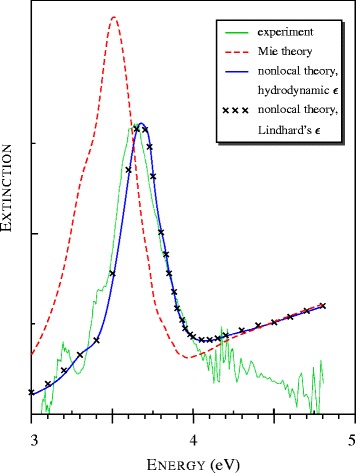
Spectra of light extinction by silver nanometer-sized particles observed in [15] and calculated with local and nonlocal models. All theoretical spectra are presented in common relative units

Let us compare predictions of the classical and generalized Lorentz-Mie theories with experimental data. In [15], Hilger, Tenfelde, and Kreibig studied extinction spectra of silver nanoparticles deposited on dielectric surfaces. In the first stage of the study, the researchers generated beams of silver particles with mean diameters of 2, 3.5, and 4 nm, determined the particle size distribution for one of the beams, recorded extinction spectra, and estimated parameter *A*=0.25 of the phenomenological formula *Γ*=*Γ*_b_+*A* *v*_F_/*R*, where *Γ*_b_ is the bulk-metal relaxation rate, for silver spheres in vacuum. First, we calculated the extinction spectra for a beam of silver spheres with the mean diameter 〈*D*〉=2 nm and experimental size distribution which spans the region from *D*=1 to *D*=4 nm. Our theory contains no adjustable parameters. In order to define the dielectric functions, we used the tabulation of the refractive index of bulk silver proposed by Lynch and Hunter [16] (see Fig. 1). We also applied Eqs. (16), (21), and (22) with *ω*_p_=9.17 eV, *Γ*_b_=0.021 eV, *v*_F_=1.39×10^6^ m/s, and *A*=0.25. The results of the calculations and experimental spectrum are presented in Fig. 2.

The theoretical spectra in Fig. 2 were calculated using the Klimontovich-Silin-Lindhard and much simpler hydrodynamic dielectric functions. It is surprising that both calculations gave close results even though |*a*|>1 in the region of the plasmon resonance.

For the nanometer-sized silver spheres, the maximum in the extinction spectrum, called the Fröhlich [17], plasmon, and surface plasmon polariton (SPP) [15] resonance, is known to shift from 3.5 to 3.65 eV [18]. The nonlocal model is in excellent agreement with the experimental data, while the local (Mie) theory gives the maximum at *ω*≃3.5 eV (see Fig. 2 and Table 1).

**Table 1:** Resonant frequencies and widths of the dipolar plasmon resonance of single silver particles and particles’ beam

Size	*ω*_m_ (eV)				*Δ* *ω* (eV)			
	THEORY							
	Local		Nonlocal		Local		Nonlocal	
	*A*=0	*A*=0.25	*A*=0	*A*=0.25	*A*=0	*A*=0.25	*A*=0	*A*=0.25
*D*=4nm	3.50	3.51	3.61	3.61	0.23	0.36	0.20	0.26
*D*=3 nm	3.50	3.51	3.65	3.65	0.23	0.39	0.18	0.26
*D*=2 nm	3.51	3.51	3.74	3.74	0.23	0.44	0.15	0.28
*D*=1 nm	3.51	3.51	–	–	0.23	0.58	–	–
〈*D*〉=2 nm	3.50	3.51	3.68	3.68	0.23	0.42	0.25	0.33
	EXPERIMENT							
〈*D*〉=2 nm	3.65				0.33			

The calculation of the blue shift of the plasmon resonance can be supported by the following consideration. In the electrostatic approximation, only *b*_1_ contributes to the extinction cross section *Q*_ext_ and Eq. (30) can be simplified by using the following approximations

32 $$ \frac{k_{2} R\,\psi_{l}^{\prime}(k_{2} R)}{\psi_{l}(k_{2} R)}\simeq l+1;\,\,\, \frac{k_{1} R\,\zeta_{l}^{\prime}(k_{1} R)}{\zeta_{l}(k_{1} R)} \simeq -\,l.  $$

Thus, *Q*_ext_ has a maximum at

33 $$  \Re [2\,(1+\delta_{1})\,\epsilon_{1}+ \epsilon_{2}]=0.  $$

The obtained condition (33) takes into account excitation of the **L** modes (by the term *δ*_1_) and, therefore, differs from the Fröhlich resonance condition [17]:.

34 $$ \Re (2\,\epsilon_{1}+ \epsilon_{2})=0.  $$

In experiment [15], the peak frequencies *ω*_m_ and resonance widths *Δ* *ω* of the extinction spectra were almost independent of 〈*D*〉. This feature of *Δ* *ω* seems to disagree with the classical Mie theory. Really, the local theory predicts broadening of the plasmon resonances with the decrease in *D* (at *A*=0.25) as shown in Table 1. At the same time, the nonlocal theory gives approximately equal resonance widths but different peak positions. Superposition of the contributions from all particles gives the value of *Δ* *ω* which are in remarkable agreement with the experimental data. It is interesting that the nonlocal theory predicts a broadening of the plasmon resonance of a beam even at *A*=0.

At *ω*>4 eV, the smooth theoretical curves in Fig. 2 lie higher than the mash of narrow closely located experimental peaks. The interband absorption dominates in this spectral range as can be confirmed by Fig. 1. The observed peculiarities of the spectrum are likely to be a consequence of a transition from the continuum bands to a discrete level structure. Such a quantum-size effect was discovered earlier in a study of the optical properties of gold nanospheres [19]. When the silver-sphere size was increased to 〈*D*〉=3.5 nm, the absorption first increased relative to the maximum and formed a plateau with a series of small equidistant dips. Then, the absorption slightly decreased at 〈*D*〉=4 nm.

**Fig. 3:**
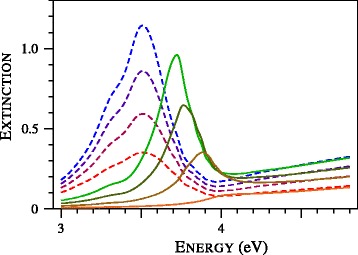
Normalized extinction cross section of silver particles with diameters of 2.2, 1.8, 1.4, and 1.0 nm calculated with local (*dashed lines*) and nonlocal (*solid curves*) Mie theories. The smaller the particle, the lower the curve. All theoretical cross sections are presented in common relative units

**Fig. 4:**
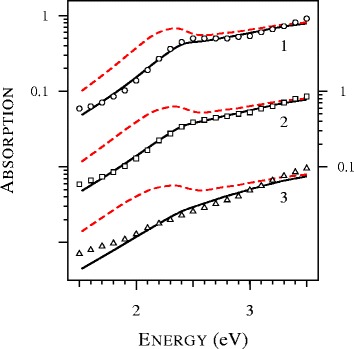
Absorption spectra calculated with the local (*dashed lines*) and nonlocal (*solid lines*) Mie theory and experimental data (*dots*) extracted from Fig. 9 of [19] for gold spheres with *D*=2.5, 2.1, and 1.7 nm in toluene (curves 1 and *circles*, curves 2 and *squares*, and curves 3 and *triangles*, respectively). All theoretical spectra are normalized to unity at 4.12 eV and displaced vertically

In order to study the formation of the blue wing of the plasmon resonance, we calculated the extinction spectra of ultra-tin silver particles and presented them in Fig. 3. A remarkable feature in Fig. 3 is complete vanishing of the plasmon resonance at *D*=1 nm. Earlier, this effect was observed in the experimental study of gold nanospheres [19]. In particular, in Fig. 9 of [19], the experimental spectra of particles with diameters of 1.7, 1.9, 2.0, 2.1, 2.3, and 2.5 nanometers were compared with the spectra calculated with the local Mie theory. The agreement was poor, failing to describe the broadening of the plasmon resonance and its position [19]. The attempts to improve the fit by varying the size of the particles and modifications of the dielectric functions were not successive. According to the authors of [19], the observed abnormally wide or depressed collective oscillation band resists to be fitted with the proposed corrections of the local Mie theory. As can be seen from Fig. 4, the situation changes dramatically if the nonlocal Mie theory is applied. Note that we used no adjustable parameters. The tabulation of the complex refractive index by Johnson and Christy [20] was used to determine the dielectric function of gold. Other parameters, including *A*=1 and refractive index of toluene (1.37) were taken from [19].

### Wave Numbers of the Longitudinal Waves

The longitudinal modes differ from the transverse ones by much higher values of the wavenumbers. For example, for the calculations presented in Fig. 2, the real part of $k_{2}^{\mathrm {L}}$ corresponds to the spatial period $\Lambda =2 \pi /\Re k_{2}^{\mathrm {L}}$ decreasing from 9 to 2 nm at *ω* increasing from 3 to 4 eV. In this *ω* interval, the absolute value of the ratio $k_{2}^{\mathrm {L}}/k_{2}$ decreased from 130 to 100 and the parameter *δ* of Eq. (27) decreased from 0.01 to 0.005 at *θ*_1_=*π*/4. We conclude, therefore, that excitation of the L waves at a flat silver surface can be neglected. However, the **L** modes have been found to be of importance in nanometer-sized silver clusters.

A replacement of the term $-\,\omega ^{2}_{\mathrm {p}}/(\omega ^{2}+i \Gamma \omega)$ in Eq. (16) by *ε* ^T^−*ε*_g_ according to Eq. (21) allows us to rewrite the dispersion Eq. (15) in the following form

35 $$  1+\frac{3}{5}\,a^{2}+\frac{3}{7}\,a^{4}+\frac{3}{9}\,a^{6}+\dots= \frac{1}{1-\epsilon^{\mathrm{T}}/\epsilon_{\mathrm{g}}}.  $$

In the simplest case of *ε*_g_=1 and *Γ*=0, Eq. (35) predicts that metal is transparent for both transverse and L waves at *ω*>*ω*_p_ but both *k* ^L^ and *k* ^T^ are complex at *ω*<*ω*_p_.

If solid is transparent, a longitudinal wave can be excited under oblique incidence of a p-polarized wave on a plane surface. There are several distinct features of this effect. First, the longitudinal waves can be generated at a flat surface, whereas special efforts should be made to excite the surface plasmon polaritons [4, 5]. Secondly, in the interference pattern, the electromagnetic-field intensity is modulated not along but perpendicular to the interface. Therefore, voids can appear in planes parallel to the surface due to spallation of the solid. According to the definition of *ω*_p_, condition *ω*>*ω*_p_ can be met in solids (for example, semiconductors) with a low density of the current carriers. We do not examine this case here because the formula of *ε* ^L^ was derived for degenerate electron gas.

## Conclusions

In order to define a spatially dependent dielectric function, all previous researchers considered interaction of matter with a plane electromagnetic wave. This approach is not constructive and rigorous in nano-optics when the field is localized in a cavity and the boundary conditions must be somehow taken into account. We have solved this problem by calculating the response of the medium on an electric field that satisfies the vector Helmholtz equation. The derived spatially dispersive dielectric function depends on the square of the wavenumber, a parameter of the Helmholtz equation, but not the wave vector of a plane wave.

We report the Fresnel reflection coefficients modified due to excitation of the longitudinal waves in metals. Similar generalization was made earlier for the Mie coefficients. Herein, the theory has been verified with simulation of light extinction by nanometer-sized silver and gold clusters. The calculated shift from 3.5 to 3.65 eV and the width of the surface plasmon resonance of the silver particles’ beam are in excellent agreement with the experimental data. In addition, the nonlocal model explains the vanishing of the plasmon resonance of golden spheres with diameters of about 2 nm. It is important that L wave can be excited on a flat surface by a plane incident wave. This is the main difference of the plasmon polaritons from the surface plasmon polaritons.

The properties of the electromagnetic oscillations in metals have been examined. It has been found that the absolute values of the wavenumbers of the longitudinal waves are much larger than those of the transverse waves. For example, in silver at a photon energy of 3.5 eV, the ratio of the absolute values of the wavenumbers is equal to 130. There, the real part of the wavenumber of the longitudinal wave corresponds to a wavelength of 7 nm. The large difference in the wavenumbers prevents excitation of the L waves at a planar surface. However, the **L** modes have been shown to be excited in silver and gold nanometer-sized particles.
